# Bird species diversity in Altai riparian landscapes: Wood cover plays a key role for avian abundance

**DOI:** 10.1002/ece3.5493

**Published:** 2019-08-20

**Authors:** Na Li, Yuehua Sun, Hongjun Chu, Yingjie Qi, Lan Zhu, Xiaoge Ping, Chunwang Li, Zhigang Jiang

**Affiliations:** ^1^ Key Laboratory of Animal Ecology and Conservation Biology, Institute of Zoology Chinese Academy of Sciences Beijing China; ^2^ University of Chinese Academy of Sciences Beijing China; ^3^ Mt. Kalamaili Nature Reserve Xinjiang China; ^4^ Xinjiang University Xinjiang China

**Keywords:** beta diversity, bird, conservation, riparian landscape, species richness

## Abstract

**Aims:**

We aim to understand bird richness and variation in species composition (beta diversity) along a 630 km riparian landscape in the Altai Mountains of China and to test whether vegetation cover is the main explanation of species diversity.

**Methods:**

We selected nine regions along a gradient of natural vegetation change. Bird surveys and environmental measurements were conducted at 10 points in each of the nine regions. We collected environmental land cover variables such as wood cover (area proportion of trees and shrubs with saplings in habitats; here trees are woody plant with a single trunk and higher than 3 m, shrubs and saplings are distinguished from trees by their multiple trunks and shorter height) and tree cover, and two climate factors which were Annual Mean Temperature (AMT) and Annual Precipitation (AP). We used Liner Regression Models to explore the correlation between bird species richness and environmental variables. We used Sørensen's dissimilarity index to measure birds' beta diversity, and quantified the contribution of environmental variables to this pattern using a Canonical Correspondence Analysis (CCA).

**Results:**

Wood cover was the strongest predictor of overall, insectivore, and omnivore bird richness. Regions with wood cover contained more bird species. Beta diversity was overall high in the studied regions, and turnover components occupied a major part of beta diversity. Wood cover and AP were significant predictors of bird species composition explaining 33.24% of bird beta diversity together.

**Conclusions:**

Wood vegetation including trees, shrubs, and saplings, rather than only trees, contains high bird richness. High beta diversity suggests that expansion of the existing nature reserves is needed in the riparian landscapes to capture the variation in bird species composition. Thus all wood cover in the overall riparian landscapes of Altai Mountains should be protected from farming and grazing to improve bird conservation outcomes.

**OPEN RESEARCH BADGES:**

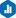

This article has earned an Open Data Badge for making publicly available the digitally‐shareable data necessary to reproduce the reported results. The data is available at Raw bird data in this study: osf.io/78qcw; Raw environment data: osf.io/qr5cw.

## 1 INTRODUCTION

Riparian landscapes are among the most diverse and complex terrestrial habitats which contain a variety of biota (Naiman, Decamps, & Pollock, 1993). Due to unique vegetation characteristics compared with neighboring areas, riparian landscapes maintain distinct bird assemblages and higher richness (Bennett, Nimmo, & Radford, 2014; Berges, Moore, Isenhart, & Schultz, 2010; Palmer & Bennett, 2006; Palmer, Fitzsimons, Antos, & White, 2008; Pennington, Hansel, & Blair, 2008). Previous research has been focusing on the impacts of local habitat characteristics in riparian regions on species diversity (Liang et al., 2018; Martin, McIntyre, Catterall, & Possingham, 2006), with many studies showing that bird richness was positively related to the width of riparian habitats (Hillman et al., 2016; Nimmo, Haslem, Radford, Hall, & Bennett, 2016; Shirley & Smith, 2005; Zimbres, Peres, & Machado, 2017) and the highly heterogeneous vegetation structure (Farley, Ellis, Stuart, & Scott, 1994; Gomez, Rivera, Politi, & Ruggera, 2016; Lynn et al., 1998), in which trees and/or woods are usually the most important. Complexity of landscape contexts in riparian strips attracts more bird species (Berduc, Lorenzon, & Beltzer, 2015; Nimmo et al., 2016; Terraube et al., 2016; Woinarski et al., 2000), whereas livestock grazing has negative effects on bird species richness (Ammon & Stacey, 1997; Dobkin, Rich, & Pyle, 1998; Jansen & Robertson, 2001; Martin & Possingham, 2005; Nelson, Gray, & Evans, 2011). It is also known that bird species richness is strongly associated with climate factors, such as precipitation and temperature (Li et al., 2013). Luke et al. (2019) found that riparian landscapes are beneficial to biodiversity and ecosystem functions in tropical areas. Riparian habitats in arid environments where the surrounding areas lack a variety of resources for species survival may be more critical for biodiversity conservation. Finding out which environmental factors affect bird species richness is important to maintain diversity in these areas.

Foraging guilds often respond to environmental conditions differently (Balestrieri et al., 2015). Recent studies have demonstrated population declines of long‐distance migrant insectivore birds in forest habitats (Gregory et al., 2007; Vickery et al., 2014). Avian foraging guilds with plant‐based diet (e.g., herbivore and omnivore) are more closely associated with tree species composition (Hasui, Gomes, & Silva, 2007), channel slope is the most influential variable in insectivore richness (Sullivan, Watzin, & Keeton, 2007), and lower gradient stream supports greater insectivore richness (Iwata, Nakano, & Murakami, 2003; Sullivan et al., 2007). Particularly, it is known that frugivore bird richness is correlated with plant diversity at a broad scale (Kissling, Rahbek, & Böhning‐Gaese, 2007). Factors associated with species richness may be various in different food guilds, thus it is important to identify environmental factors that affect bird richness of various food guilds in riparian regions. Guild‐specific guidelines are necessary to improve protection effectiveness in maintaining bird diversity.

Beta diversity represents the change in species composition among sites (Whittaker, 1960, 1972) and may help to optimize conservation networks, thus has been widely studied since 2000 (Anderson et al., 2011). Beta diversity is composed of two processes: turnover and nestedness (Baselga, 2007, 2010; Harrison, Ross, & Lawton, 1992). Habitats with high beta diversity, especially high turnover component are often considered a high priority for conservation value. Many studies examined the contribution of turnover and nestedness to beta diversity process in mountain landscapes (Blake & Loiselle, 2000; Jankowski, Ciecka, Meyer, & Rabenold, 2009; Jankowski et al., 2013; Patterson, Stotz, Solari, Fitzpatrick, & Pacheco, 1998), on islands (Si, Baselga, & Ding, 2015) and in riparian landscapes (Berduc et al., 2015). It is reported that the more heterogeneous the environment, the higher beta diversity is. Thus understanding bird beta diversity and its underlying mechanisms in riparian landscapes could be helpful to set up conservation areas.

In this context, we investigated bird diversity (species richness and beta diversity) and tested the role of land cover and climate factors in shaping bird richness and composition in riparian landscapes. We hypothesized that bird diversity could be explained by change in vegetation cover. We also aim to identify habitats of particular conservation value due to species richness and beta diversity along the riparian landscapes of Altai Mountains, China.

## 2 MATERIALS AND METHODS

### 2.1 Study area

**Figure 1:**
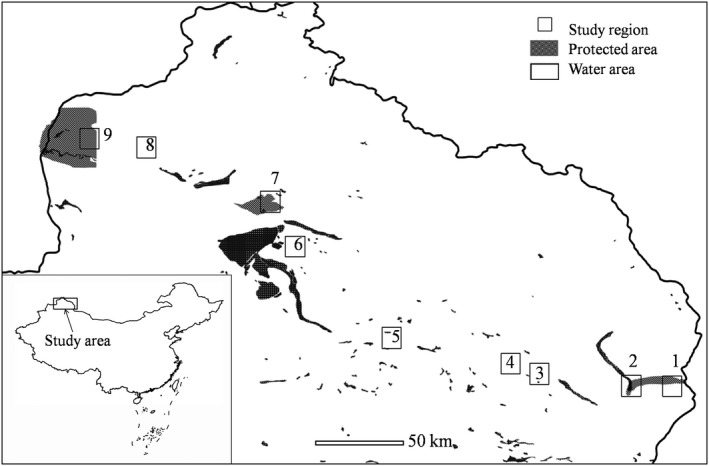
Location of the nine study regions in the riparian landscapes of Altai Mountains, China

Our study area is riparian landscapes located between 480 ~ 1,800 m above sea level on the southwest slope of Altai Mountains in China (E 90.70°–85.52°, N 45.29°–48.42°; Figure 1). The riparian landscapes are nourished by headwaters of the Irtysh River, which originates from the Altai Mountains, flows from east to west and joins Ulungur River in the piedmont plain. The length of the riparian landscapes is about 633 km and the area is about 5.273 × 10^4^ km^2^. The riparian landscapes as well as the surrounding desert landscapes have temperate arid climate with mountainous influence: mean annual temperature is 4.2°C, mean annual precipitation is 100 mm and mean annual evaporation is 2,000 mm (Zhang, Zhou, Ye, & Bin, 2016). Natural soil types in the riparian landscapes are cultivated meadow soil, bench land moist soil, meadow brown calcic soil, and semifixed eolian sandy soil. The riparian landscapes have mosaics of natural forest patches, shrubs, wetland, desert (main soil type is brown desert soil), bare land, and agroforestry land and are dominated by agriculture, which is usually for growing watermelons, corns, and sunflowers. The dominant wood species in original riparian forest that distribute along the sides of the river valley with sufficient water conditions are salicaceous woods, including poplars, aspens, and shrub willows. The area with natural forest is <10% of the riparian landscapes. Farmlands (1.5 × 10^3^ km^2^) and grass lands (3.6 × 10^4^ km^2^) distribute in forest habitats. Nomadic residents use the grasslands as pastures; grasses are mowed in August every year and stored as winter forage. Surroundings of the riparian landscapes are desert landscapes (38 × 10^4^ km^2^) including the Junggar Basin in the south. Common plant species in this desert landscapes are sagebrush (*Artemisia* spp.) and reed grass (*Calamagrostis* spp.), which are usually shorter than 0.2 m with a coverage of 10% ~ 20% and are distinct from the riparian landscapes.

### 2.2 Bird survey

In 2015 we selected nine separated regions (*n* = 9) with natural forest patches as survey sites along the riparian landscapes (Figure 1), based on presurvey in 2014. We established 10 bird survey points in each of the nine regions. The first sample points were randomly set and thereafter a systematic scheme was followed with distance larger than 300 m between adjacent points. Only nine sample points were set in region two due to topographical structure. Five minutes counts at each point were repeated monthly in summer (June to August) in the morning (from sunrise to 4 hr after sunrise) and afternoon (from 3 hr before dusk to dusk) in days without heavy winds or rain, and visiting time was reversed to avoid diurnal changes in bird activity. Region three, four, and seven were visited in year 2015, and others were in 2016. Six points in region eight and five points in region one were also visited in 2015. Positions of all points were recorded with a global position system (GPS) receiver with accuracy of 5 m. At each point, within a 100 m radius circle, all species seen or heard were identified, and spotted distances were recorded. Bird species recorded at each site during the morning and afternoon surveys were summed. Only the larger number of individuals of each species between the morning and afternoon surveys was used to avoid double‐counting the same individuals (Jankowski et al., 2009; Sullivan et al., 2007). Nocturnal surveys were not undertaken and therefore species active at night (e.g., owls) were poorly sampled. Immature birds were not taken in the count and barn swallow (*Hirundo rustica*) and bird species depending on aquatic habitats were not included in this study. The first author conducted bird counts, accompanied by two field assistants. The first author has 5 years' experience in bird watching. Both the first author and field assistants were trained to identify local bird species and common tree species in year 2013 and 2014 before data collection.

### 2.3 Environmental variables

At each sample point, we collected the following land cover types: trees, woods, grasslands, farmlands, wetlands, bare lands, deserts, roads (footpaths between fields without gravel or cement), and human settlements (nomadic shelters built up by woods and rocks). The wood cover included trees, shrubs, and young saplings. Shrubs and young saplings are shorter than 3 m and multiple stems, which were distinguished from trees. From the center of each sample point, three radial 100 m lines were established, separated by 120°. On each of the lines, within a 1 m‐wide strip, the different land cover types were visually assessed, and the average value on three radial lines was calculated as the final area proportion in a sample point. We also collected climate variables including Annual Mean Temperature (AMT) and Annual Precipitation (AP) from WorldClim (Hijmans, Cameron, Parra, Jones, & Jarvis, 2005). AMT and AP values of each sample point were extracted using DIVA‐GIS (version 7.5.0.0), and averaged values from year 2000 to 2005 were used in the analysis.

### 2.4 Avian foraging guild classification

Bird species were assigned to different foraging guilds based on field observations and the literature (e.g., Ma, 2011). We classified bird species into foraging guilds such as herbivores, carnivores, omnivores or insectivores. Piscivorous species (e.g., kingfisher) were not included in our foraging guild analyses.

### 2.5 Data analysis

We rarefied sample points to nine in each region to even the field effort and used Mantel Correlogram (Legendre & Legendre, 2012) to test spatial autocorrelations of bird diversity. We then explored the relationship between bird species richness and each environmental variable using Ordinary Least Squares (OLS) models for all birds and for each foraging guild. All variables were standardized (mean = 0 and standard deviation = 1) to make the regression coefficients comparable and to improve fit during model selection. We also explored the combination of variables that most associated with bird species richness using Multiple Linear Regression (MLR). We first tested the correlation between every pair of environment variables using Pearson Correlation Test. There were strong correlations between wood cover and AP (*r* = −.73), wood cover and AMT (*r* = −.83), crop cover and wetland cover (*r* = .92). We removed AP, AMT and wetland cover from the MLR process to decrease collinearity. Then the best‐fit model was selected from all possible combinations of the eight variables, guided by the lowest Akaike Information Criterion value (AIC_w_).

Following Baselga (2010), we distinguished change in species composition by partitioning total beta diversity (Sørensen's dissimilarity index, *β*_sor_) into turnover component (Simpson's dissimilarity, *β*_sim_) and nestedness‐resultant component (*β*_nes_). Bird species presence–absence data within regions were summarized, and a region × species matrix (9 rows × 83 columns) was created. Dissimilarity coefficients were calculated in the whole riparian landscape (across nine regions) and between every pair of adjacent regions (total eight region pairs from the upriver to the down river). Relationships between bird species composition and environmental variables were evaluated by Canonical Correspondence Analysis (CCA; Legendre & Legendre, 2012). CCA was performed on the region × species matrix and a region × environmental matrix (9 rows × 11 columns). We used the Forward Selection procedure (Blanchet, Legendre, & Borcard, 2008) to reduce collinearity among variables. Likewise, orthogonal ordination axes were extracted to represent the contribution of individual environmental variables, and help identifying the most parsimonious model. The variance explained by ordination axes was examined. Permutation tests (Monte Carlo Method with 999 random runs) were used to examine the overall significance of the models. All analyses were performed in R 3.1.3 using packages *vegan* and *betapart* (R Development Core Team, 2010).

There was slight difference in the bird species composition and species richness in region eight and region one between 2015 and 2016, due to gain or loss of one or two species. To test whether the temporal bias in sampling would affect out results, we randomly chose one bird record from 2 years in 2015 and 2016 and conducted the above Mantel Correlogram, OLS, beta diversity and CCA analyses. We repeated the procedure several times, and results of the analyses were robust. Here, we only reported the results using bird records in 2015 for region three, four, and seven, and in 2016 for other regions.

## 3 RESULTS

### 3.1 Bird species richness and associated environmental factors

**Figure 2:**
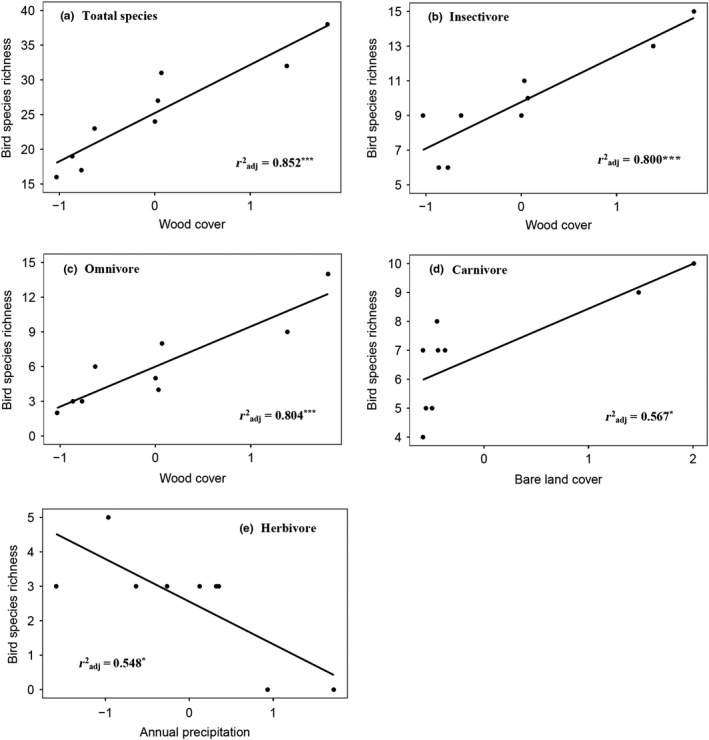
Relationships between bird species richness and the most associated variables: (a) total species, (b) insectivore and (c) omnivore are most associated with wood cover, (d) carnivore is most associated with bare land cover and (e) herbivore is most associated with annual precipitation. The OLS linear fits and adjusted *r* ^2^ are shown. Statistically significant *p*‐values less than .05, .01 and .001 are indicated as *, ** and ***, respectively

A total of 3,072 detections of 83 bird species were recorded across all 89 points. Of the 83 bird species, 26 were residents, 55 were summer migrants. Likewise, there were 32 of insectivores which formed the largest guild whereas the herbivores formed the smallest guild with nine species (Table 1). Though only nine sample points were set in region two, bird species richness was the highest. The rarefied data results were robust with original data analysis, thus we only reported the statistical analysis based on the original data. There was no spatial autocorrelation in bird diversity. The univariate OLS models showed that wood cover was the variable that positively influenced all bird species (Coefficient = 6.948, *t*‐value = 6.866, Adjusted *r* ^2^ = .852, Standard Error = 1.012, Degrees of Freedom = 7, *p*‐value = .0002), and variables that have negative effect on all bird richness were farmland cover (Coef = −5.625, *t *= −3.049, $r_{\text{adj}}^{2}$ = .509, *SE* = 1.845, *df* = 7, *p* = .018) and wetland cover (Coef = −5.502, *t *= −2.901, $r_{\text{adj}}^{2}$ = .481, *SE* = 1.897, *df* = 7, *p* = .023). For bird insectivores, species richness was positively correlated with wood cover (Coef = 2.679, *t* = 5.757, $r_{\text{adj}}^{2}$ = .801, *SE* = 0.465, *df* = 7, *p* = .0007) and negatively correlated with AMT (Coef = −2.386, *t *= −3.643, $r_{\text{adj}}^{2}$ = .605, *SE* = 0.654, *df* = 7, *p* = .008). Wood cover was the only influential variable for omnivore birds and had positive effect on species richness (Coef = 3.467, *t* = 5.822, $r_{\text{adj}}^{2}$ = .804, *SE* = 0.595, *df* = 7, *p* = .0006). For the carnivore guild, species richness was only positively correlated with the bare land cover (Coef = 1.549, *t* = 3.392, $r_{\text{adj}}^{2}$ = .567, *SE* = 0.457, *df* = 7, *p* = .012). The herbivore richness was positively correlated with wood cover (Coef = 1.076, *t* = 2.432, $r_{\text{adj}}^{2}$ = .381, *SE* = 0.442, *df* = 7, *p* = .045) and negatively correlated with AP (Coef = −1.237, *t *= −3.276, $r_{\text{adj}}^{2}$ = .548, *SE* = 0.378, *df* = 7, *p* = .014). Our results show that wood cover positively affected bird species richness except for the carnivore guild and was the most correlated variable for all birds, omnivores, and insectivores (Figure 2). The variables that had the largest influence in carnivores and herbivores were bare land cover and AP, respectively (Figure 2). MLR showed that no multivariate model performed better in explaining the variation of bird species richness for insectivores and herbivores than the OLS models (Table 2). Wood cover was consistently included in the best‐fit model except for bird carnivores (Table 2).

**Table 1:** Elevation range, number of survey points, individual detections, total species richness and species richness of individual foraging guild within and across nine riparian regions from upriver to downriver

Riparian region	Elevation range(m)	No. of points	No. of individuals detected	Total species richness	Species richness of Insectivores	Species richness of Omnivores	Species richness of Carnivores	Species richness of Herbivores
1	1,091–1,130	10	289	32	13	9	7	3
2	1,047–1,098	9	292	38	15	14	4	5
3	833–871	10	184	24	9	5	7	3
4	814–821	10	359	27	11	4	9	3
5	641–669	10	451	17	6	3	5	3
6	471–571	10	417	19	6	3	7	3
7	475–481	10	232	31	10	8	10	3
8	450–570	10	721	23	9	6	8	0
9	448–571	10	127	16	9	2	5	0
Total	448–1,130	89	3,072	83	32	21	21	9

**Table 2:** The best‐fit Multiple Linear Regression models of species richness for overall and each bird foraging guild against the eight variables

Bird foraging guild	WOD	FAR	BAR	ROA	SET	GRA	TRE	DES	$r_{\text{adj}}^{2}$	*p*
Overall species									.967	.0046
Insectivores									.801	.0006
Omnivores									.923	.0043
Carnivores									.724	.0519
Herbivores									.381	.0452

Note

Each column is a different variable (WOD, FAR, BAR, ROA, SET, GRA, TRE, and DES are area proportion of woods, farmlands, bare lands, roads, human settlements, grasslands, trees and deserts, respectively). Gray cell indicates that the variable was included in the particular combination (each row). Adjusted *r* ^2^ and statistically significant *p*‐value of each model were listed.

### 3.2 Beta diversity and environmental explanation

Most bird species showed narrow spatial distribution. On average, individual bird species was detected in 2.7 regions. Only two bird species, black‐eared Kite (*Milvus lineatus*) and Eurasian tree sparrow (*Passer montanus*), were detected in all regions. We found 16 species (19.3%) occurring in more than five of the regions, while 34 species (41.0%) were restricted to a single region. For individual guilds, the proportion of species occurring in more than half of the regions was 18.8% (eight species) for insectivores, 14.3% (three species) for omnivores, 23.8% (five species) for carnivores, and 22.2% (two species) for herbivores, respectively.

**Figure 3:**
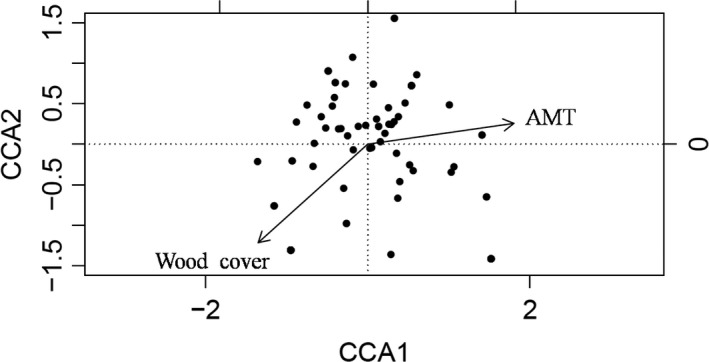
The plot of bird species (points, *n* = 83) and environmental variables (lines with arrows) from Canonical Correspondence Analysis. The environmental variables were wood cover and AMT (annual mean temperature). Longer line means a more contribution of an environmental variable to ordination axes. Smaller angle between line and axes represents a stronger correlation between environmental variable and axes. The closer the distance between a species point and an environmental variable arrow, the stronger the affect of the variable to distribution pattern of the species is. The direction of arrow represents negative or positive of the correlation between environmental variable and axes. The strength of wood cover to CCA1 is −0.740 and to CCA2 is −0.667, and strength of AMT to CCA1 is 0.989, to CCA2 is 0.141. Total 33.24% variation of species composition was explained, with 16.72% contribution from CCA1 and 16.51% from CCA2

High beta diversity and turnover component were found in the study regions. In the whole riparian landscape, the overall beta diversity was .78, and the turnover component was .71. Between adjacent riparian regions, *β*_sor_ varied from .43 to .68, and *β*_sim_ made up a large component of change in species composition compared with *β*_nes_ across adjacent regions (Table 3). Simplified model by CCA included only wood cover and AMT and explained 33.2% of bird composition variation (*p* = .002; Figure 3) with the first ordination axis (CCA1) explained 16.72% and the second (CCA2) explained 16.51% of the variance.

**Table 3:** Beta diversity (*β*_sor_) and its nestedness (*β*_nes_) and turnover component (*β*_sim_) calculated between every pair of adjacent regions from upriver to down river

Adjacent pairs	*β*_sor_	*β*_nes_	*β*_sim_	*β*_sim_/*β*_sor_
1	.43	.05	.38	.87
2	.68	.09	.58	.86
3	.49	.03	.46	.93
4	.46	.16	.29	.65
5	.50	.03	.47	.94
6	.56	.14	.42	.75
7	.56	.08	.48	.86
8	.44	.12	.31	.72

Note

The ratios of turnover component to total beta diversity (*β*_sim_/*β*_sor_) showed that turnover component contributed a main proportion to total beta diversity.

## 4 DISCUSSION

We found that wood cover which includes both trees and shrubs mixed with saplings positively affected bird species richness in the riparian landscape of Altai Mountains. Beta diversity was high in the whole landscape, and turnover was the main component of beta diversity. Best‐fit model by CCA included wood cover and AMT, which explained up to 33.2% variation of bird species composition. Our results suggest that, wood cover, including trees as well as shrubs and young saplings, and not only trees, should be protected for conserving high bird diversity in the whole riparian landscape.

### 4.1 Bird species richness

Riparian landscapes are crucial habitats for birds in arid areas. In general, bird species richness and abundance increase with the area and width of woody vegetation (Berges et al., 2010; Deschenes, Belanger, & Giroux, 2003). Numerous studies have demonstrated that tall shrubby and wooded strips contained greater bird diversity and abundance than the other types of vegetation (Deschenes et al., 2003; Lynn et al., 1998; Powell & Steidl, 2015), and riparian bushes are important for bird conservation in tropical arid areas (Arizmendi et al., 2008). Martin et al. (2006) found that riparian areas with tree layer exhibited high bird richness and were dominated by small‐bodied insectivores. Our results are consistent with that of the former researchers, and we find that rather than tall trees, habitat patches containing both trees and shrubs often have a greater vegetation complexity which may create additional niches for bird species. Ouin et al. (2015) claimed that reducing understory vegetation or dead wood might negatively affect insectivores. Jankowski et al. (2013) found vegetation structure was an important predictor for insectivores and omnivores which include insects as major dietary component. In our study, insectivores occupied a large component of overall bird species, insectivores and omnivores were most closely associated with woods in their habitats, and the abundance of these two foraging guilds might benefit both from abundance of terrestrial insects feeding on riparian vegetation and emergent stream insects (Murakami & Nakano, 2002; Nakano & Murakami, 2001; Sullivan et al., 2007), though shoreline birds, not included in this study, may feed on the larvae of aquatic insects.

### 4.2 Beta diversity

High beta diversity was found between bird communities in our study regions, and nestedness component contributed much less than turnover to the dissimilarity in bird compositions. Birds found in less diverse regions were not subsets of the species in more diverse regions. The environmental variables explained only 33.24% of species composition variation among regions, suggesting that there are other environmental factors to be accounted for. For instance, Jankowski et al. (2013) found that tree species composition was a more important predictor than vegetation structure for all foraging guilds. An increasing number of studies prove that species occurrence at any place might depend not only on the characteristics at that site but also on characteristics of the larger landscape (e.g., Lee & Rotenberry, 2015; Nelson et al., 2011; Nimmo et al., 2016). Habitats may be appealing to different species due to variations in habitat structure such as the combination of tree species, shrubs and grass (Berges et al., 2010). Best, Bergin, and Freemark (2001) found few bird species were residents of row crop fields; many used them only for feeding and this was more likely to happen when crop fields were associated with an adjacent wooded habitat. Berges et al. (2010) found crop and pasture sites had less suitable habitat for many bird species, presumably due to a lack of habitat structure in the form of trees, shrubs, or tall grasses. In our study, many avian species recorded on the crop site or pasture site were often birds live in small size patches of woods dispersed on the agriculture or grassland matrix, such as azure tit (*Parus cyanus*), great tit (*parus major*), European bee‐eater (*Merops apiaster*), spotted flycatcher (*Muscicapa striata*) and Eurasian collared dove (*Streptopelia decaocto*); or fly‐catching species roaming overhead, such as northern house martin (*Delichon urbica*), sand martin (*Riparia riparia*), and fork‐tailed swift (*Apus pacificus*); or birds known to prefer open habitat and usually perch on electricity lines to prey, for instance, European roller (*Coracias garrulus*) and lesser kestrel (*Falco naumanni*). Consequently, though woody species composition and larger‐scale environmental factors were not considered in our models, the function of wood cover in predicting changes in bird composition could be reasonable.

## 5 CONCLUSIONS

Riparian areas are among the most threatened landscapes (Groffman et al., 2003). Human societies have historically settled down near streams and rivers for access to water, food, needs of irrigation, transportation and water power for industry (Pennington et al., 2008). Meanwhile, riparian areas provide high vegetation connectivity and corridors for birds to move through the landscapes (Sekercioglu et al., 2015). Riparian landscapes in the Altai Mountains are strip oases with shifting mosaic of habitat patches. Natural vegetation is crucial for both resident and migrant birds and harbors high bird species diversity. The woodlands in the riparian area are clear cut for farmlands, logged for fuels, or used as pastures. In particular, shrubs and young saplings are used for grazing and mowing. Consequently the riparian areas are being converted to shrub lands, grasslands, or farmlands. Maintenance of woody vegetation is vital in supporting a wide variety of bird species. Hence, conservation strategies that maximize preservation of the whole riparian areas, rather than particular areas, should be employed. Mixed woods landscape, not only tall trees, but also shrubs and young saplings, should be protected.

## CONFLICT OF INTEREST

There is no conflict of interest exist.

## AUTHORS' CONTRIBUTIONS

N. L., Z. J. and Y. S. conceived the ideas and designed methodology; N. L., H. C., Y. Q. and C. L. collected the data; N. L. and L. Z. analyzed the data; N. L., Z. J., X. P. and C. L. led the writing of the manuscript. All authors contributed to the drafts.

## Data Availability

Raw data are provided in Dryad repository (https://doi.org/10.5061/dryad.3m66bd1).
